# Flavonoids: A complementary approach to conventional therapy of COVID-19?

**DOI:** 10.1007/s11101-020-09720-6

**Published:** 2020-09-18

**Authors:** Julia Solnier, Johannes-Paul Fladerer

**Affiliations:** Institute of Pharmaceutical Sciences, Department of Pharmacognosy, University of Graz, Universitätsplatz 4, 8010 Graz, Austria

**Keywords:** Flavonoids, Coronaviruses, Positive-sense RNA viruses, COVID-19, SARS-CoV, SARS-CoV-2, MERS-CoV

## Abstract

COVID-19, the highly contagious novel disease caused by SARS-CoV-2, has become a major international concern as it has spread quickly all over the globe. However, scientific knowledge and therapeutic treatment options for this new coronavirus remain limited. Although previous outbreaks of human coronaviruses (CoVs) such as SARS and MERS stimulated research, there are, to date, no antiviral therapeutics available that specifically target these kinds of viruses. Natural compounds with a great diversity of chemical structures may provide an alternative approach for the discovery of new antivirals. In fact, numerous flavonoids were found to have antiviral effects against SARS-and MERS-CoV by mainly inhibiting the enzymes 3-chymotrypsin-like protease (3CLpro) and papain-like protease (PLpro). In this review, we specifically focused on the search for flavonoids, polyphenolic compounds, which are proven to be effective against human CoVs. We therefore summarized and analyzed the latest progress in research to identify flavonoids for antiviral therapy and proposed strategies for future work on medicinal plants against coronaviruses such as SARS-CoV-2. We discovered quercetin, herbacetin, and isobavachalcone as the most promising flavonoids with anti-CoV potential.

## Introduction

Historically, viral diseases have always emerged and posed major issues to public health. Several viral outbreaks—such as the severe acute respiratory syndrome coronavirus (SARS-CoV) in 2002–2003, the H1N1 influenza virus in 2009, and the Middle East respiratory syndrome coronavirus (MERS-CoV) in 2012—have caused serious global health concerns in recent years (Cascella et al. 2020). Over the past 50 years, there has been a noticeable increase in the emergence of different novel coronaviruses responsible for a wide range of human and veterinary diseases (Fehr and Perlman 2015). Most recently, a new viral epidemic with numerous cases of unexplained low respiratory tract infections occured in Wuhan, Hubei Province, China, as it was first reported to the World Health Organization (WHO) on 31 December 2019 (World Health Organization 2020b). The novel virus strain was identified as the Severe Acute Respiratory Syndrome Coronavirus 2 (SARS-CoV-2) triggering coronavirus disease 2019 (COVID-19) (He et al. 2020a). On 11 March 2020, the WHO declared COVID-19 a pandemic (World Health Organization 2020a**).**

Coronaviruses (CoVs) are highly diverse, enveloped, positive-sense, single-stranded RNA viruses (+ ssRNA) (He et al. 2020a), which constitute the biggest group of viruses within the *Nidovirales* order, containing the largest genomes for RNA viruses (Fehr and Perlman 2015).

In total, about 30 CoVs have so far been recognized to be able to infect different species, including humans, mammals, fowl, and other animals (Li et al. 2020). Among them, seven human CoVs, belonging to the alpha‐ and beta‐CoVs groups (Li et al. 2020), have been identified as being capable of infecting humans, including 229E, NL63, OC43 HKU1, MERS-CoV, SARS-CoV and the novel SARS-CoV-2 (Centers for Disease Control and Prevention 2020; Fehr and Perlman 2015; Zhu et al. 2020). The name ‘coronavirus’ is inspired by its most defining feature: the club-shaped spikes projecting from the surface of the virion. The spikes sticking out of the envelope’s surface give the virus the appearance of a crown (Fehr and Perlman 2015).

The nucleocapsids of CoVs, enclosing the genomic RNA, are helically symmetrical. This is in fact unusual for positive-sense RNA viruses, and far more common for negative-sense RNA viruses (Fehr and Perlman 2015). The two overlapping open-reading-frames (ORF1a and ORF1b) of SARS, translated into the viral enzymes 3C-like protease (3CLpro) and papain-like protease (PLpro), which are vital for virus multiplication, constitute approximately two-thirds of the genome (Adedeji et al. 2012). The other one-third of the genome encodes structural proteins of the virus, such as the spike (S), envelope (E), membrane (M) and nucleocapsid (N) proteins (Adedeji et al. 2012). The interaction between the S-protein and the receptor is the primary determinant for a coronavirus to infect a host species (Lim et al. 2016). To date, it is known that SARS-CoV attaches to its receptor angiotensin-converting enzyme 2 (ACE2), while MERS-CoV was found to bind to dipeptidyl-peptidase 4 (DPP4) in order to penetrate human cells (Fehr and Perlman 2015). So far, it has been observed that the new coronavirus SARS-CoV-2 behaves much like SARS by using the same entry mechanism to human cells (Rabi et al. 2020) and sharing a 79.5% genome sequence identity to SARS-CoV (Yang et al. 2020; Zhou et al. 2020). Several studies have demonstrated that novel SARS-CoV-2 likely binds to the human ACE2 receptor, but with a higher affinity than the original SARS virus strain (Gurwitz 2020; Letko et al. 2020; Rabi et al. 2020; Wrapp et al. 2020; Xu et al. 2020).

**Fig. 1:**
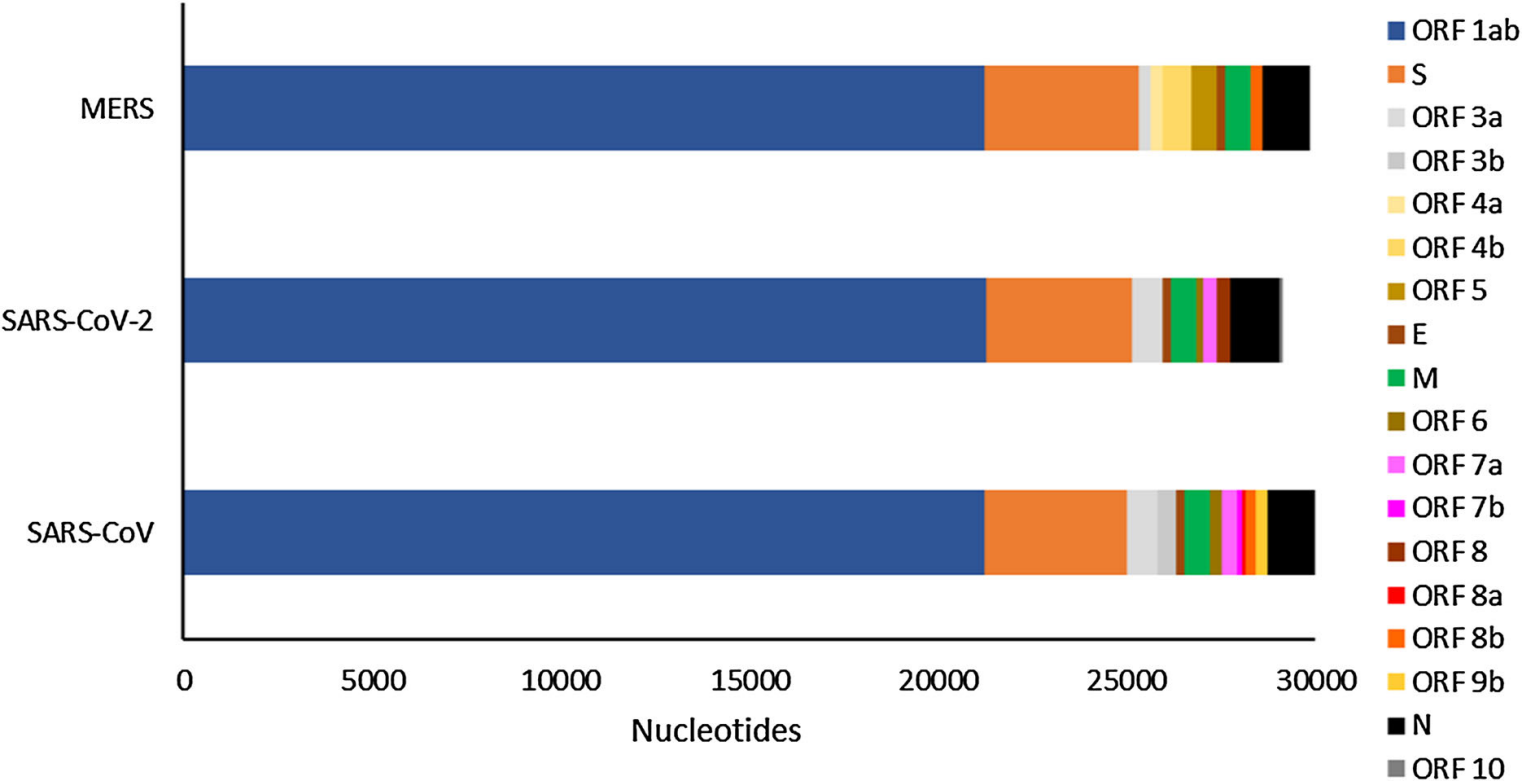
Comparison of the genomic structures of MERS, SARS-CoV and SARS-CoV-2. The data are extracted from the original publications in GenBank. Especially SARS-CoV and SARS-CoV-2 show high similarity. ORF = open-reading-frame; spike (S), envelope (E), membrane (M) and nucleocapsid (N) proteins

Genetic data demonstrated that SARS-CoV-2 possesses overlapping open-reading-frames (ORF1a and ORF1b) similar to those of SARS- and MERS-CoV (Fig. 1), translated into the viral enzymes 3CLpro and PLpro. SARS- and SARS-CoV-2 share a 3CLpro sequence similarity of 96%, and a PLpro sequence identity of 83% (McKee et al. 2020). Therefore, 3CLpro and PLpro present two key targets for the development of anti-SARS-CoV-2 therapeutics as both are crucial for viral replication; and they share significant homology with proteases of several other related coronaviruses (Goetz et al. 2007).

## Therapeutic approach

Even though the SARS and MERS outbreaks stimulated research on human CoVs, there are, to date, no antiviral therapeutics available that specifically target these viruses (Rabaan et al. 2020). Several potential vaccines, including recombinant attenuated viruses, live virus vectors, or individual viral proteins expressed from DNA plasmids, have been developed for SARS-CoV; however, none of them are yet approved for clinical use (Fehr and Perlman 2015). There are several reports which propose potential drugs, although their clinical efficacy has not yet been confirmed for SARS-CoV-2 infection and COVID-19 disease. These drugs include: chloroquine, lopinavir/ritonavir, remdesivir, umifenovir, nucleoside analogs, neuraminidase inhibitors, DNA synthesis inhibitors (e.g. tenofovir disoproxil, and lamivudine), ACE2-based peptides, novel vinylsulfone protease inhibitors, teicoplanin, 3-chymotrypsin-like protease (3CLpro)- and papain-like protease (PLpro) inhibitors (Lai et al. 2020; McKee et al. 2020). To date, the application of remdesivir appears to be the most promising strategy for COVID-19 (Lai et al. 2020). In preclinical studies, it has been shown that remdesivir (GS5734)—an inhibitor of RNA polymerase with in-vitro activity which was used against various RNA viruses, including Ebola—could be effective for both prophylaxis and therapy against human CoV infections (Gordon et al. 2020). Alpha-interferon and lopinavir/ritonavir have also been suggested for the treatment of CoVs (Cascella et al. 2020).

## Flavonoids and their antiviral potential against coronaviruses

Since therapy options for coronaviruses, such as for COVID-19, comprise only preventive and supportive measures, natural products may have a fundamental role in supportive and prophylaxis treatments, and present an alternative approach for CoV-management. Flavonoids form the largest group of polyphenolic compounds in higher plants (Nileeka Balasuriya and Vasantha Rupasinghe 2011), with more than 9000 structures identified (Wang et al. 2018). They represent an important class of plant secondary metabolites, widely distributed throughout the plant kingdom (Wang et al. 2018).

**Fig. 2:**
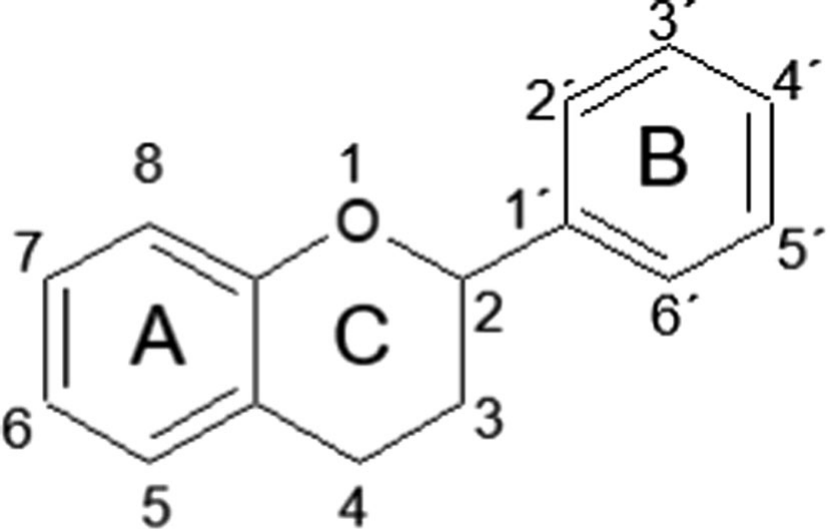
Flavan structure

Flavonoids are categorised into several subgroups, which include chalcones, flavanes, flavanols, flavanones, flavanonols, flavones, flavonols, isoflavones or catechins, and procyanidins, all of them consisting of a common flavan (2-phenylchroman) basic structure (Fig. 2). These polyphenolic substrates perform a series of protective functions in the human body. Many of them are bioactive compounds capable of interfering with nucleic acid or proteins, meaning that they have diverse pharmacological properties (Panche et al. 2016). It has been reported that flavones and catechins appear to be the most powerful antioxidants, preventing the effects of reactive oxygen species in the body (Nijveldt et al. 2001; Panche et al. 2016). There are numerous studies highlighting the broad range of biological activities of flavonoids, including antioxidant (D’Amelia et al. 2018), anti-cancer (LeJeune et al. 2015), antimicrobial (Abreu et al. 2017; Solnier et al. 2020), antiviral (Wang et al. 1998), and anti-inflammatory (Catarino et al. 2016) activities (Panche et al. 2016).

More importantly, various flavonoids have been found to inhibit different targets of coronaviruses SARS and MERS (Yang et al. 2020), such as blocking the enzymatic activites of viral proteases like 3-chymotrypsin-like protease (3CLpro), papain-like protease (PLpro) and helicase or interfering with spike (S) proteins. A few flavonoids were shown to suppress the activity of angiotensin-converting enzyme (ACE) (Guerrero et al. 2012; Nileeka Balasuriya and Vasantha Rupasinghe 2011), which not only plays an important role in cardiovascular diseases such as in hypertension, but may also represent a key determinant in viral infections, and pneumonia (Actis-Goretta et al. 2006).

Another important issue presents the anti-inflammatory potential of flavonoids in viral diseases, such as activating and stimulating the host immune response to viral infections (Dong et al. 2014), but also suppressing overwhelming inflammatory reactions, which are often associated with a higher mortality rate of SARS-CoV-2 infections (McKee et al. 2020). For instance, some flavonoids have been reported to interfere with the activation of NLRP3 inflammasome (Lim et al. 2018) which upregulates the production of inflammatory cytokines, and thus can cause respiratory distress syndrome that frequently occurs within SARS coronavirus diseases (Chen et al. 2019), and SARS-CoV-2 infections (Shah 2020).

However, most studies on flavonoids—showing their numerous health-beneficial properties—are conducted in vitro based on the fact that these polyphenolic compounds often deal with low bioavailability, little stability and poor distribution when tested in vivo using animal and/or human cell models (Nileeka Balasuriya and Vasantha Rupasinghe 2011). There are different strategies reported to enhance these functions, such as the introduction of structural modifications (Srinivas 2009), absorption enhancers or nanotechnology (Ajazuddin and Saraf 2010; Zhao et al. 2019).

The aim of this review is to evaluate the information on flavonoids as possible leads for developing therapeutics to treat SARS-CoV-2. Therefore, we searched PUBMED for studies reporting on the effects of flavonoids on human coronaviruses. Most of the studies targeted the enzymatic activities of the viral proteases 3CL and PL in vitro using FRET (fluorescence resonance energy transfer)-based methods. 3CL and PL proteases represent valuable targets for the development of anti-coronaviral drugs, as these proteins are essential for the viral transcription and replication complex of all coronaviruses (Anand et al. 2003; Chen et al. 2005b; Park et al. 2016), translated from two open reading frames ORF1a and ORF1b which are found in SARS-, SARS-2-, and MERS-CoV (Chuck et al. 2011; Lin et al. 2004). According to the literature, we were able to identify 47 flavonoids which might present potential agents to treat SARS-CoV-2 (Table 1). Detailed analysis of their structure–activity relationships, has ultimately led to the identification of the three most promising compounds with broad-spectrum antiviral activity.

**Table 1:** Chemical structures of flavonoids tested against CoVs

	Structure	Substance	R1	R2	R3	R4	R5	R6	R7	R8
Chalcone		4-hydroxyderricin	H	OH	OH	CH_2_CH=C(CH_3_)_2_	OCH_3_	H	H	
		4-hydroxyisolonchocarpin	H	OH	OH	H	OC(CH3)_2_CH=CHR^6^	R^5^	H	
		4′-O-methylbavachalcone	H	OCH_3_	OH	H	OCH_3_	CH_2_CH=C(CH_3_)_2_	H	
		Broussochalcone A	OH	OH	OH	H	OH	CH_2_CH=C(CH_3_)_2_	H	
		Broussochalcone B	H	OH	OH	H	OH	CH_2_CH=C(CH_3_)_2_	H	
		Helichrysetin	H	OH	OCH_3_	H	OH	H	OH	
		Isobavachalcone	H	OH	OH	CH_2_CH=C(CH_3_)_2_	OH	H	H	
		Xanthoangelol	H	OH	OH	CH_2_CH=C(CH_3_)_2_	OCH_3_	H	OH	
		Xanthoangelol B	H	OH	OH	CH_2_CH=C(CH3)CH2CH2CHOHC(CH_3_)=CH_2_	OH	H	H	
		Xanthoangelol D	H	OH	OH	CH_2_CH=C(CH3)CH2CH2CH=O	OH	H	H	
		Xanthoangelol E	H	OH	OH	CH_2_CHOOHC(CH_3_)=CH_2_	OCH_3_	H	H	
		Xanthoangelol F	H	OH	OH	CH_2_CH=C(CH_3_)_2_	OCH_3_	H	H	
		Xanthoangelol G	H	OH	OH	CH_2_CH=C(CH3)CH2CH2CH=C(CH_3_)_2_	OCH_3_	H	H	
Flavane		3′-(3-methylbut-2-enyl)-3′,4,7-trihydroxyflavane	H	H	H	H	OH-CH_2_CH=C(CH_3_)_2_	OH	OH	
		Broussoflavan A	CH_2_CH=C(CH_3_)_2_	OH	OC(CH_3_)_2_CHOHCHOHR^4^	R^3^	H	H	OH	
		Kazinol A	CH_2_CH=C(CH_3_)_2_	OH	OH	CH_2_CH=C(CH_3_)_2_	H	H	OH	
		Kazinol B	CH_2_CH=C(CH_3_)_2_	OH	OC(CH_3_)_2_CH=CHR^4^	R^3^	H	H	OH	
Flavanole		Epigallocatechin gallate	OH	OH	OH	C=O-3,-4,-5-trihydroxyPh	OH	OH		
		Gallocatechin gallate				Konfomer				
Flavanone		Bavachinin	H	OH	H	CH_2_CH=C(CH_3_)_2_	OCH_3_			
		Hesperetin	OH	OCH_3_	OH	H	OH			
Flavanonole		Ampelopsin	OH	OH	OH	OH	OH			
Flavone		Amentoflavone	3´,8´´- Biapigenin							
		Apigenin	H	OH	OH	H	OH			
		Luteolin	OH	OH	OH	H	OH			
		Pectolinarin	H	OCH_3_	OH	OCH_3_	(6-O-(6-deoxy-alpha-L-mannopyranosyl)-beta-D-glucopyranosyl)oxy			
		Rhoifolin	H	OH	OH	H	O-rhamnoglucoside			
		Scutellarein	H	OH	OH	OH	OH			
Flavonole		7-O-arylmethylquercetin—derivatives	H	OH	OH	H	OH	H	OCH2Ph (3′'-Cl, 3′'-CN, or 4′'-Cl)	H
		Herbacetin	H	OH	H	H	OH	H	OH	OH
		Kaempferol	H	OH	H	H	OH	H	OH	H
		Myricetin	OH	OH	OH	H	OH	H	OH	H
		Papyriflavonol A	CH_2_CH=C(CH_3_)_2_	OH	OH	H	OH	CH_2_CH=C(CH_3_)_2_	OH	H
		Quercetin	OH	OH	H	H	OH	H	OH	H
		Quercetin 3-β-d-glucoside	OH	OH	H	Glucoside	OH	H	OH	H
		Quercetin-3-β-galactoside	OH	OH	H	Galactoside	OH	H	OH	H
		Tomentin A	OH	OH	H	H	OH	CH_2_CH_2_C-OR^7^-CH_3_-(CH_2_)_3_C(CH_3_)_2_-OH	R^6^	H
		Tomentin B	OH	OCH_3_	H	H	OH	CH_2_CH_2_C-OR^7^-CH_3_-(CH_2_)_3_C(CH_3_)_2_-OH	R^6^	H
		Tomentin C	OH	OCH_3_	OCH_3_	H	OH	CH_2_CH_2_C-OR^7^-CH_3_-(CH_2_)_3_C(CH_3_)_2_-OH	R^6^	H
		Tomentin D	OCH_3_	OH	OCH_3_	H	OH	CH_2_CH_2_C-OR^7^-CH_3_-(CH_2_)_3_C(CH_3_)_2_-OH	R^6^	H
		Tomentin E	OCH_3_	OH	H	H	OH	CH_2_CH_2_C-OR^7^-CH_3_-(CH_2_)_3_C(CH_3_)_2_-OH	R^6^	H
Isoflavone		Corylifol A	CH_2_CH=CCH_3_CH2CH2 CH=C(CH_3_)_2_	OH	OH	H				
		Daidzein	H	OH	OH	H				
		Neobavaisoflavone	CH_2_CH=C(CH_3_)_2_	OH	OH	H				
		Puerarin	H	OH	OH	β -D-glucopyranose				
Procyanidine		Procyanidin A2	H	OH	OH	R^5^	R^4^			
		Procyanidin B1	OH	OH	H	H	H			

## Major targets of flavonoids against CoVs

The various studies have focused primarily on the interference of flavonoids with some viral proteases such as 3CL and PL of SARS- and MERS-CoV by using common enzymatic, fluorogenic (FRET)-based methods and molecular docking studies, as summarized in Table 2. 3CLpro and PLpro are both key targets as they process several viral polyproteins which are involved in the replication and transcription of the genomic RNA within host cells (McKee et al. 2020). Further, they share significant homology with viral proteases of several other coronaviruses—especially with those of SARS-, SARS-2-, and MERS-CoV (Wu et al. 2020).

**Table 2:** In-vitro antiviral activity of flavonoids against enzymes of SARS- and MERS-CoV

	Substance	SARS 3CLpro IC50[µM]	SARS PLpro IC50[µM]	MERS 3CLpro IC50[µM]	MERS PLpro IC50[µM]	Method	Literature	Plant
Chalcone	4-hydroxyderricin	81.4(cf), 50.8(cb)	26			FRET	Park et al. (2016)	*Angelica keiskei*
	4-hydroxyisolonchocarpin	202.7	35.4	193.7	171.6	FRET	Park et al. (2017)	*Broussonetia papyrifera*
	4′-O-methylbavachalcone		10.1			FRET	Kim et al. (2014)	*Cullen corylifolia*
	Broussochalcone A	88.1	9.2	36.2	42.1	FRET	Park et al. (2017)	*Broussonetia papyrifera*
	Broussochalcone B	57.8	11.6	27.9	112.9	FRET	Park et al. (2017)	*Broussonetia papyrifera*
	Helichrysetin			67.04		FRET	Jo et al. (2019)	
	Isobavachalcone	39.4(cf), 11.9(cb)	13, 7.3	35.85		FRET	Jo et al. (2019), Park et al. (2016), Kim et al. (2016)	*Angelica keiskei, Cullen corylifolia*
	Xanthoangelol	38.4(cf), 5.8(cb)	11.7			FRET	Park et al. (2016)	*Angelica keiskei*
	Xanthoangelol B	22.2(cf), 8.6(cb)	11.7			FRET	Park et al. (2016)	*Angelica keiskei*
	Xanthoangelol D	26.6(cf), 9.3(cb)	19.3			FRET	Park et al. (2016)	*Angelica keiskei*
	Xanthoangelol E	11.4(cf), 7.1(cb)	1.2			FRET	Park et al. (2016)	*Angelica keiskei*
	Xanthoangelol F	34.1(cf), 32.6(cb)	5.6			FRET	Park et al. (2016)	*Angelica keiskei*
	Xanthoangelol G	129.8(cf), NA(cb)	46.4			FRET	Park et al. (2016)	*Angelica keiskei*
Flavane	3′-(3-methylbut-2-enyl)-3′,4,7-trihydroxyflavane	30.2	35.8	34.7	48.8	FRET	Park et al. (2017)	*Broussonetia papyrifera*
	Broussoflavan A	92.4	20.4	125.7	49.1	FRET	Park et al. (2017)	*Broussonetia papyrifera*
	Kazinol A	92.4	66.2	NA	88.5	FRET	Park et al. (2017)	*Broussonetia papyrifera*
	Kazinol B	233.3	31.4	NA	94.9	FRET	Park et al. (2017)	*Broussonetia papyrifera*
Flavanole	Epigallocatechin gallate	73				FRET	Jo et al. (2020), Nguyen et al. (2012)	
	Gallocatechin gallate	47				FRET	Jo et al. (2020), Nguyen et al. (2012)	
Flavanone	Bavachinin		38.4			FRET	Kim et al. (2014)	*Cullen corylifolia*
	Hesperetin	60(cf), 8.3(cb)				CA	Jo et al. (2019), Lin et al. (2005)	*Isatis tinctoria*
Flavanonole	Ampelopsin	364				FRET	Nguyen et al. (2012)	
Flavone	Amentoflavone	8.3				FRET	Jo et al. (2020), Ryu et al. (2010a)	*Torreya nucifera*
	Apigenin	280.0				FRET	Jo et al. (2020), Ryu et al. (2010a)	
	Luteolin	20				FRET	Jo et al. (2020), Ryu et al. (2010a)	
	Pectolinarin	37.78				FRET	Jo et al. (2020)	
	Rhoifolin	27.45				FRET	Jo et al. (2020)	
	Scutellarein	IC50=0.86 µM against nsP13				FRET	Keum and Jeong (2012), Yu et al. (2012)	
Flavonole	7-O-arylmethylquercetin—derivatives (3′'-Cl, 3′'-CN, and 4′'-Cl)	IC50=5.2, 2.7, 4.1 μM against NTPase and helicase of SARS-CoV				FRET	Park et al. (2012)	
	Herbacetin	33.17		40.59		FRET	Jo et al. (2019), Jo et al. (2020)	
	Kaempferol	116.3	16.3	35.3	206.6	FRET	Jo et al. (2020), Schwarz et al. (2014)	
	Myricetin	IC50=2.71 µM against nsP13				FRET	Keum and Jeong (2012), Yu et al. (2012)	
	Papyriflavonol A	103.6	3.7	64.5	112.5	FRET	Park et al. (2017)	*Broussonetia papyrifera*
	Quercetin	52.7 (73) (23.8)	8.6	34.8	NA	FRET	Chen et al. (2004), Jo et al. (2020), Lee et al. (2009), Lin et al. (2005), Nguyen et al. (2012), Ryu et al. (2010a)	
	Quercetin 3-β-d-glucoside			37.03		FRET	Jo et al. (2019)	
	Quercetin-3-β-galactoside	128.8, 42.79 µM	51.9	68.0	129.4	FRET	Chen et al. (2006)	
	Tomentin A		6.2			FRET	Cho et al. (2013)	*Paulownia tomentosa*
	Tomentin B		6.1			FRET	Cho et al. (2013)	*Paulownia tomentosa*
	Tomentin C		11.6			FRET	Cho et al. (2013)	*Paulownia tomentosa*
	Tomentin D		12.5			FRET	Cho et al. (2013)	*Paulownia tomentosa*
	Tomentin E		5			FRET	Cho et al. (2013)	*Paulownia tomentosa*
Isoflavone	Corylifol A		32.3			FRET	Kim et al. (2014)	*Cullen corylifolia*
	Daidzein	351, 26.8(cf), NA(cb)				FRET, CA	Jo et al. (2020), Nguyen et al. (2012)	
	Neobavaisoflavone		18.3			FRET	Kim et al. (2014)	*Cullen corylifolia*
	Puerarin	381				FRET	Jo et al. (2020), Nguyen et al. (2012)	
Procyanidine	Procyanidin A2	29.9 (wild type SARS-CoV)				PRA	Zhuang et al. (2009)	*Cinnamomum cassia*
	Procyanidin B1	41.3 (wild type SARS-CoV)				PRA	Zhuang et al. (2009)	*Cinnamomum cassia*

IC50 is the inhibitory concentration of compound required to cause 50% inhibition of virus

FRET, fluorescence resonance energy transfer; PRA, Plaque reduction assay; CA, Cleavage assay; NA, No activity; (cf) cell-free; (cb) cell-based

Plant names are checked with theplantlist.org

Besides 3CL protease, papain-like protease has been the focus of numerous studies on the development of chemotherapeutic drugs against CoV-induced diseases. PLpro is not only responsible for processing viral polyproteins, but is also involved in deubiquitination (cleaving ubiquitin chains) and deISGylation, which represent relevant factors in the host immune response to viruses (Cho et al. 2013). By removing ubiquitin and ISG15 proteins from host cell proteins, the innate host immune response is likely to be compromised (Ratia et al. 2014; Xian et al. 2020). PLpro of both SARS- and MERS-CoV has been shown to possess deubiquitinating activities (Park et al. 2017). Structural and functional analyses of SARS-CoV PLpro have revealed that PLpro is homologous to human deubiquitinating enzymes capable of cleaving ubiquitin and ubiquitin-like proteins (Park et al. 2016) and may, therefore, have an important part in the virus life cycle. Hence, antiviral therapeutics targeting PLpro could also prevent the antagonist activities of PLpro on host innate immune response (Ratia et al. 2014).

In FRET-based assays, the proteolytic activity is detected through cleavage of a fluorogenic peptide and measuring the increase in fluorescence intensity by continuously monitoring the reaction (Park et al. 2017). FRET is a non-destructive method, widely exploited to study protein interactions (Margineanu et al. 2016), and is also applied to detect signals in living cells (Huang et al. 2020). While the cell-free FRET assays can provide easy and fast access to proteins including some difficult targets (Sierecki et al. 2013), the cell-based FRET methods enable in situ analysis of a variety of biological targets and protein–protein interactions in a more natural environment (Silverman et al. 1998). The studies of Park et al. (2017), Lin et al. (2005) and others could prove that flavonoids tested in cell-based assays generated higher activity against 3CL and PL, than excerting unspecific activity against other enzymes. Furthermore, the unspecific aggregation of proteins often caused by flavonoids could be reduced significantly by the addition of Triton X without affecting anti-coronaviral activity in these studies.

In a number of studies molecular docking technology was used for screening purposes. Despite the valuable use of this method to rapidly discover novel inhibitors of SARS-CoV-2, there are some disadvantages which include low accuracy and high rate of false-positive results (Wang et al. 2020). However, compounds which are identified through such virtual screening methods can be further examined in high-throughput enzymatic in-vitro assays, followed by more in-depth in vivo investigations, such as studies on efficacy and toxicity.

In general, the combination of docking studies and bioactivity assays can improve screening accuracy by providing supportive data.

## Inhibitors of 3CLpro and PLpro

### Chalcones

Chalcones isolated from *Angelica keiskei* were shown to inhibit both SARS-CoV proteases PLpro and 3CLpro in enzymatic, FRET-based (Table 2) and molecular docking studies (Park et al. 2016). Inhibition was achieved through a competitive manner against 3CLpro, and a non-competitive mode against PLpro (Park et al. 2016). Xanthoangelol E, an alkylated chalcone substituted with a –OOH group (Table 1), exerted the most relevant inhibition against 3CLpro (IC_50_ = 11.4 and 7.1 µM) and PLpro (IC_50_ = 1.2 µM) using fluorogenic methods (Park et al. 2016) (Table 2). Analyses of the relationships of the alkylated chalcones (Tables 1 and 2) against 3CLpro showed that xanthoangelol E substituted with 2-perhydroxyl-3-methyl-3-butenyl (PMB) group was more effective than xanthoangelol D with a 2-hydroxy-3- methyl-3-butenyl group (IC_50_ = 26.6 and 9.3 µM). Similarly, the hydroxyl group of the A-ring caused apparently higher activity than when substituted with a methyl group. Comparing xanthoangelol D and 4-hydroxyderricin, it seemed that the substitution of the A-ring with a 2-hydroxy-3-methyl-3-butenyl group (xanthoangelol D, Table 1) generated higher inhibition than when substituted with the dimethylallyl group (4-hydroxyderricin: IC_50_ = 81.4 and 50.8 µM, Table 2) (Park et al. 2016). Based on the results it can be assumed that a perhydroxyl group on the substituted hemiterpene might be crucial for enzyme binding and may affect conformational stabilization of the polyhydroxylated chain through intramolecular hydrogen bonding (Park et al. 2016).

In general, the bioactivity of highly potent compounds against SARS PLpro apparently depended on modifications in their molecular structure, such as prenylation in the A and B ring and methylation. For instance, the prenyl moiety of the 3′-prenylated chalcones isobavachalcone and xanthoangelol E led to considerable inhibition against SARS PLpro (IC_50_ = 7.3 and 1.2 µM), highlighting the importance of the hydrophobic substituent. Further, it is generally assumed that prenylation of flavonoids may enhance their bioactivity and bioavailability (Grienke et al. 2016; Mukai 2018), presumably by altering lipophilicity and thereby affinity for membrane targets (Shen et al. 2012). Thus, chemical modifications of flavonoids, such as the introduction of hydrophobic substituents, may also demonstrate a reasonable strategy to enhance their antiviral activity against SARS-CoV-2.

Some flavonoids isolated from *Broussonetia papyrifera* have been proposed as useful lead-compounds for the development of anti-CoV agents against SARS and MERS (Park et al. 2017) and therefore, might be also considered to treat SARS-CoV-2. Among them, broussochalcone A, broussochalcone B, and 4-hydroxyisolonchocarpin derived from *B. papyrifera* showed proteolytic activity against both proteases 3CL and PL of SARS- and MERS-CoV (Table 2) through non-competitive inhibition. As Table 2 demonstrates, the compounds showed generally higher inhibitory potential against SARS-CoV PLpro than when tested against the other viral proteases using fluorogenic methods, which is likely related to genomic variations in the single amino acid sequences.

Broussochalcone B was found to be the most effective substrate for inhibiting 3CLpro of MERS-CoV (IC_50_ = 27.9 μM) in the study of Park et al. (2017). In addition to inhibitory effects on cysteine proteases, the compounds isolated from *B. papyrifera* caused inhibition against alpha-glucosidase (Park et al. 2017). Inhibition of glycosidases, especially of alpha-glucosidases, affects maturation, transport, secretion, and the functioning of glycoproteins which can enhance cell—cell and/or cell—virus recognition processes (Ryu et al. 2010b). Therefore, it plays a relevant role in the treatment of viral infections, but also in other diseases such as diabetes mellitus type 2, and cancer (Ryu et al. 2010b). In accordance with a previous study (Ryu et al. 2010b), the alpha-glucosidase inhibitory activity of the substrates from *B. papyrifera* depends on the number and positions of prenyl groups present in the molecule (Park et al. 2017).

Kim et al. (2014) could demonstrate that isobavachalcone and 4′-O-methylbavachalcone extracted from the seeds of *Cullen corylifolia* have great inhibitory potential against SARS-CoV PLpro. Both compounds inhibited PLpro in a dose-dependent manner, with IC_50_ values of 7.3 and 10.1 µM (Table 2) (Kim et al. 2014). Isobavachalcone (2′,4,4′‐trihydroxy‐3′‐(3‐methyl‐2‐butenyl) chalcone) also showed inhibitory activity against MERS-CoV 3CLpro (IC_50_ = 35.85 μM, Table 2), when tested within the screening of a flavonoid library using a fluorogenic (FRET-based) method (Jo et al. 2019).

Based on the results summarized in Table 2, the chalcones isobavachalcone, 4′-O-methylbavachalcone, broussochalcone A and B, including xanthoangelols, can be considered as prominent inhibitory compounds of SARS-CoV PLpro (IC_50_ ≤ 12.5 µM, Table 2). For comparison, Báez-Santos et al. reviewed numerous inhibitors of SARS-CoV PLpro (Báez-Santos et al. 2015) reporting tanshinones and diarylheptanoids as successful inhibitors of SARS-CoV PLpro, with IC50 values of tanshinones ranging from 0.8 to 30.0 µM (Báez-Santos et al. 2015). Disulfiram has been suggested to be a putative inhibitor of SARS-and MERS-CoV PLpro (IC_50_ = 24.1 µM and 14.6 µM) (Lin et al. 2018).

Regarding the broad-spectrum activity of chalcones, isobavachalcone presents the major compound in this group − with a good scaffold to bind with proteases of both SARS- and MERS‐CoV. Hence, further in-vitro investigations with isobavachalcone on viral proteins of SARS-CoV-2, as well as some pharmacological studies in-vivo regarding toxicity and bioavailability of the compound, might be a valuable approach in the field of research for COVID-19. There are a few plant sources of isobavachalcone other than those mentioned in Table 2, as it is abundantly found in species belonging to plant families Fabaceae (e.g., *Anthyllis hermanniae, Glycyrrhiza glabra, Glycyrrhiza uralensis, Sophora prostrata*) and Moraceae (e.g., *Dorstenia poinsettifolia, Dorstenia turbinata, Maclura tinctoria, Treculia acuminata*) (Kuete and Sandjo 2012).

### Flavanes and flavanols

The flavanes 3′-(3-methylbut-2-enyl)-3′’,4,7-trihydroxyflavane, broussoflavan A, kazinol A and kazinol B isolated from *Broussonetia papyrifera* interfered with viral proteases 3CL and PL of both SARS- and MERS-CoV (Table 2) through non-competitive inhibition (Park et al. 2017). Among all flavanes tested, 3′-(3-methylbut-2-enyl)-3′,4,7-trihydroxyflavane, a C5-alkyl group (prenyl)-substituted flavan (Table 1), exhibited the most potent inhibition against SARS- and MERS-CoV 3CLpro (IC_50_ = 30.2 μM and 34.7 μM). Considering the structure–activity relationships of flavonoids from *B. papyrifera* (Tables 1 and 2), it was observed that molecules having a C4-OH group and saturated chromenone with a dihydroxy group at ring C have stronger inhibitory potential than those with a closed prenyl group such as kazinol B (IC_50_ = 233.3 μM, Table 2).

Given the results, the flavane 3′-(3-methylbut-2-enyl)-3′,4,7-trihydroxyflavane may serve as good template for continuing in-vitro experiments on viral proteases of SARS-CoV-2 due to similar efficacy against proteases of both SARS- and MERS-CoV, and the homology of proteases from the SARS-CoV-2 strain (Fig. 1).

Epigallocatechin gallate (EGCG) and gallocatechin gallate (GCG), two flavanol derivatives, demonstrated inhibitory activity against SARS-CoV 3CLpro, when tested in proteolytic (FRET-based) assays and molecular docking studies of Nguyen et al. (2012). The catechin GCG (IC_50_ = 47 µM) inhibited 3CLpro activity (Table 2) by interacting with amino acid residues in the active site pocket of 3CLpro (Nguyen et al. 2012). Analyses of structure–activity relationships of these compounds (Tables 1 and 2) suggest that the galloyl moiety at position 3-OH of EGCG and GCG (Table 1), which is absent in the other catechins, seemed to be relevant for causing 3CLpro inhibition. GCG (2S, 3R type), which is a C-2 epimeric isomer of EGCG (2R, 3R type), generated higher 3CLpro inhibition than that of EGCG (IC_50_ = 73 µM). In a following docking study, it was shown that the galloyl group of GCG, was essential for hydrogen bond interactions with the amino residues Leu141, Gly143, Ser144, and His163 of the 3CLpro active pocket site (Nguyen et al. 2012). For further studies with these compounds on proteases of SARS-CoV-2 it should be considered that different conformation and structural modifications in the molecules likely increase antiviral activity.

### Flavanones and flavanonols

In a study investigating the Chinese medicinal plant *Isatis tinctoria* root—which was used for the prevention of SARS-CoV during the SARS-outbreaks, and is known for its antiviral properties against influenza, Hepatitis A and Japanese Encephalitis infections (Lin et al. 2005; Wu et al. 1997)—hesperetin was discovered as highly potent inhibitor of SARS-CoV 3CLpro (IC_50_ = 8.3 μM) when tested in a cell-based cleavage assay (Lin et al. 2005). In comparison with other reported inhibitors of SARS-CoV 3CLpro, such as cinanserin and cinanserin hydrochloride (IC_50_ = 31 μM and 34 μM, respectively) (Chen et al. 2005a), hesperetin may present a promising lead in the development of SARS-CoV-2 3CL protease inhibitors. However, further screenings on proteolytic activity of hesperetin against PLpro might be also important. Several citrus fruits are the major plant sources of flavanones, including hesperetin—the aglycone of hesperidin.

Bavachinin, a flavanone extracted from the seeds of *Cullen corylifolia,* was found to have inhibitory potential against SARS-CoV PLpro (IC_50_ = 38.4 µM, Table 2) (Kim et al. 2014).

Amelopsin, also known as dihydromyricetin, had only weak inhibitory activity against SARS-CoV 3CLpro (IC_50_ = 364 µM, Table 2) when tested in a proteolytic (FRET-based) assay and molecular docking studies (Nguyen et al. 2012). In the same study, it was observed that the additional OH group at 5′-position in the B ring as well as the absence of 2,3-double bonds in the C-ring of amelopsin (Table 1) considerably reduced 3CLpro inhibitory activity when compared to other compounds tested, such as quercetin (IC_50_ = 73 µM, Table 2) (Nguyen et al. 2012).

### Flavones and flavonols

Amentoflavone, a biflavone isolated from *Torreya nucifera*, demonstrated a prominent inhibitor of SARS-CoV 3CLpro (IC_50_ = 8.3 μM) in a FRET-based assay (Ryu et al. 2010a). In that study, amentoflavone proved to be far more potent than the parent compound apigenin (IC_50_ = 280.8 µM), and the other flavone luteolin (IC_50_ = 20 μM), as well as the flavonol quercetin (IC_50_ = 23.8 μM). By comparing their structures (Table 1), those compounds with a C-3′-substituted hydroxyl group, such as luteolin and quercetin, excerted stronger inhibition than apigenin. Furthermore, biflavone derivatives of amentoflavone with methylation of 7-, 4′-, and 4′′′-hydroxyl groups, decreased inhibitory activity (Ryu et al. 2010a).

Based on the prominent in vitro inhibition activity of amentoflavone against 3CLpro of SARS-CoV, the compound may serve as a good starting point for further investigations on 3CLpro of SARS-CoV-2—due to 79.5% genome sequence identity (Yang et al. 2020; Zhou et al. 2020), including more than 95% similarity of 3CLpro amino acid sequences between SARS-CoV and SARS-CoV-2 (McKee et al. 2020). However, the biflavonoid should be also tested against other proteases of SARS- and MERS-CoV in order to determine a broad spectrum antiviral efficacy against SARS-CoV-2. There are numerous plant sources from which amentoflavone can be isolated, these include for instance *Gingko biloba*, *Hypericum perforatum, Lobelia chinensis*, *Polygala sibirica*, *Ranunculus ternatus*, and several plants from *Selaginella* species, such as *Selagenella tamariscina, Selaginella nipponica*, and *Selaginella pulvinata* (Yu et al. 2017).

Two other flavones, pectolinarin and rhoifolin, and the flavonol herbacetin were discovered as inhibitors of SARS-CoV 3CL enzyme when tested in proteolytic assays (IC_50_ ≤ 37.78 µM, Table 2) and induced-fit docking experiments (Jo et al. 2020). In docking studies, Jo et al. showed that the additional 8-hydroxyl group of herbacetin seems to be crucial for its high binding affinity to the polar S1 site and the hydrophobic S2 site of SARS-CoV 3CLpro (Jo et al. 2020). Furthermore, carbohydrate groups of rhoifolin and pectolinarin were responsible for the high affinity to SARS-CoV 3CLpro (Jo et al. 2020). In another screening using a FRET-based method, herbacetin (3,4′,5,7,8‐pentahydroxyflavone, Table 1) also caused inhibition against 3CLpro of MERS-CoV at an IC_50_ value of 40.59 μM (Table 2) (Jo et al. 2019). Since herbacetin shows similar inhibitory effects against both 3CL proteases of SARS- and MERS-CoV (IC_50_ = 33.17 and 40.59 μM), it may also demonstrate proteolytic activity when tested against SARS-CoV-2 3CL protease. Several 3CLpro inhibitors that have been suggested as candidates for SARS-CoV-2 3CLpro, were found to be effective against 3CLpro of both SARS- and MERS-CoV (He et al. 2020b). Herbacetin can be found, for instance, in Ephedrae herba (Hyuga et al. 2013), *Linum usitatissimum* (Qiu et al. 1999) and *Rhodiola rosea* (Péter Zomborszki et al. 2019)***.***

Papyriflavonol A, a double prenylated flavone derivative isolated from *Broussonetia papyrifera,* presents one of the most significant inhibitors of SARS PLpro at an IC_50_ value of 3.7 µM (Table 2), causing higher activity than other flavonols such as kaempferol (IC_50_ = 16.3 µM), quercetin (IC_50_ = 8.6 µM), and quercetin-beta-galactoside (IC_50_ = 51.9 µM). Similarly, geranylated flavonols from the fruits of *Paulownia tomentosa* were found to be the main group of active constituents that successfully interferes with SARS-CoV PLpro (Cho et al. 2013). Among twelve PLpro-inhibitory flavonoids isolated, five new geranylated flavonols named tomentin A-E bearing an unusual 3,4-dihydro-2*H*-pyran motif with a cyclized geranyl chain (Table 1) caused high inhibition (IC_50_ values 5–12.5 µM) within a FRET-based assay (Table 2). Prenylated and geranylated flavonoids like papyriflavonol A, and tomentins (IC_50_ ≤ 7.3 µM, Table 2) can be proposed as interesting leads for SARS-CoV-2 PLpro inhibitors due to 83% homology of PLpro sequences between SARS-CoV and SARS-CoV-2 (McKee et al. 2020).

It can be assumed that the hydrophobic substituents of prenylated flavonoids e.g., papyriflavonol A, show higher affinity to SARS-CoV PLpro than to other proteases, which might be due to certain structural differences in the protein sequences. Further, the polarity of the compounds such as an increased number of hydroxyl groups, including the addition of sugar residues in the flavone backbone (Table 1), seems to affect inhibitory potential, as demonstrated by kaempferol (IC_50_ = 16.3 µM), quercetin (IC_50_ = 8.6 µM), and quercetin-β-galactoside (IC_50_ = 51.9 µM).

Comparing quercetin with quercetin β-glycosides—the sugar moieties such as that of quercetin-3-β-galactoside, and quercetin 3-β-d-glucoside did not enhance inhibitory activity against SARS-and MERS-CoV proteases.

In molecular docking studies, quercetin-3-β-galactoside created hydrophobic interactions with residues Asn142, Glu166, Leu141 and Met165 of SARS 3CLpro (Chen et al. 2006). In particular, the residue Q189 represented a principle factor in the binding between quercetin-3-β-galactoside and SARS 3CLpro (Chen et al. 2006).

Analyses of structure–activity relationships of quercetin-3-β-galactoside derivatives suggested that hydroxyl groups on the quercetin moiety are crucial for inhibitory activity, which enable compound–target interaction through hydrogen bonding (Chen et al. 2006).

Quercetin and quercetin 3‐β‐glucoside (3,3′,4′,5,7‐pentahydroxyflavone 3‐β‐glucoside) exerted inhibition when tested against MERS 3CLpro with respective IC_50_ values of 34.8 and 37.03 μM (Table 2) (Jo et al. 2019). In a following docking study, Jo et al. showed that a rhamnose substitution instead of glucose (in quercetin 3‐β‐glucoside), results in a stronger binding with MERS‐CoV 3CLpro (Jo et al. 2019).

In general, quercetin causes a broad range of activities against proteases of SARS- and MERS-CoV (Table 2). Based on that, and the number of sources cited, such as the IC_50_ values reported in different studies, quercetin is among the most promising agents to treat SARS-CoV-2. Quercetin represents one of the most abundant dietary flavonoids that naturally occurs in a great variety of plant products (medicinal herbs, food, beverages, etc.) (Anand David et al. 2016; Pandey and Rizvi 2009). A few of these rich sources from which quercetin can be isolated include: *Allium cepa, Allium fistulosum, Asparagus officinalis, Camellia sinensis, Capparis spinosa, Coriandrum sativum, Ginkgo biloba, Hypericum perforatum, Moringa oleifeira, Punica granatum* (Howell and D'Souza 2013), *Sambucus canadensis* (Anand David et al. 2016; Li et al. 2016)*.*

### Isoflavones

The isoflavones, daidzein (IC_50_ = 351 µM) and puerarin (IC_50_ = 381 µM) lacking the B-ring in their 3-phenyl-4H-1-benzopyran-4-one backbone (Table 1), showed only weak inhibition of SARS-CoV 3CLpro, as demonstrated in Table 2. Neobavaisoflavone—structurally related to daidzein—prenylated at C3′, and corylifol A extracted from the seeds of *Cullen corylifolia*, were found to have good inhibitory potential against SARS-CoV PLpro (IC_50_ = 18.3 and 32.3 µM) (Kim et al. 2014). As mentioned before, flavonoids with lipophilic substituents like prenyl or geranyl side chains in the A or B ring (Table 1) appear to be very good scaffolds to bind with PL proteases, and may therefore be considered as templates for the development of SARS-CoV-2 PLpro inhibitors. Isoflavonoids are predominantly found in legumes; prenylated derivatives can be mainly isolated from the plant familiy Fabaceae (Šmejkal 2014).

## Inhibition of RNA-dependent RNA polymerase and Helicase

### Flavones and flavonols

A screening of 64 natural compounds against SARS-CoV helicase led to the identification of scutellarein and myricetin—two strong inhibitors of SARS nsP13 (Table 2) using an ATP hydrolysis assay (Keum and Jeong 2012; Yu et al. 2012). The non-structural proteins (nsPs), which are processed by the viral proteases PL (residing in nsp3) and 3CL (residing in nsp5), comprise the RNA-dependent RNA polymerase and the NTPase/helicase—two essential determinants for virus replication (Adedeji et al. 2012). The SARS helicase nsP13 protein has been shown to have double-strand (ds) RNA and (ds) DNA unwinding activity and can translocate along the nucleic acids by hydrolyzing ATP (Lee et al. 2010). Both compounds, the flavone scutellarein and the flavonol myricetin, successfully impaired ATPase activity of nsP13 with respective IC_50_ values of 0.86 μM and 2.71 μM (Table 2). This effect was mediated through inhibition of ATPase activity, but did not affect dsDNA-unwinding activity of SARS helicase, as it was demonstrated in a fluorometric, FRET-based, dsDNA unwinding assay of Yu et al. (2012).

Myricetin naturally occurs in high concentrations in different fruits (e.g., cranberry) (Singh et al. 2009), and vegetables such as spinach, cauliflower etc. (Sultana and Anwar 2008). Scutellarein can be found in members of the genus *Scutellaria* such as *Scutellaria baicalensis*—an important traditional Chinese plant used against diverse infections including inflammatory or respiratory diseases (Zhao et al. 2016).

Another study evaluating 24 derivatives of 7-O-aryl-methylquercetin for their antiviral activity against SARS-CoV, three derivatives with 3′'-Cl, 3′'-CN, and 4′'-Cl aromatic substituents showed selective inhibition of SARS-CoV NTPase/helicase activity by performing a FRET-based dsDNA unwinding assay (Park et al. 2012). The introduction of an arylmethyl substituent, such as 3-ClPhCH2, 3-CNPhCH2 and 4-ClPhCH2, at the position 7-OH of quercetin (Table 1), led to stronger inhibition against SARS-CoV helicase (IC_50_ values of 5.2, 2.7, 4.1 μM, respectively) than that of quercetin (IC_50_ = 8.1 µM) (Park et al. 2012).

## Inhibition of S protein

### Flavones and flavonols

The spike (S) protein of CoVs is responsible for viral entry into target cells through binding to the angiotensin-converting enzyme 2 (ACE2) receptor on host cell membranes (Zhou et al. 2020), and thus presents another important target in the anti-SARS-CoV-2 drug development. Besides the inhibitory potential of luteolin against SARS 3CL protease (IC_50_ = 20 µM, Table 2), the flavone was found to successfully impede the entry process of SARS-CoV into host cells by interfering with the S protein of SARS-CoV (EC_50_ value = 10.6 µM) in a two-step screening method combining frontal affinity chromatography-mass spectrometry (FAC/MS) and pseudotyped virus-infection assay (Yi et al. 2004). Likewise, quercetin was found to block host cell entry (EC_50_ = 83.4 µM) when tested in HIV-luciferase/SARS pseudotyped virus assay (Yi et al. 2004). The use of pseudotyped viruses is reported to be a powerful, rapid, and highly sensitive method for studying the entry process of viruses to host cells (Yi et al. 2004).

Since SARS-CoV and SARS-CoV-2 share high sequence similarity of the S proteins, these compounds may be also expected to impede the entry of SARS-CoV-2 into host cells. Furthermore, it is proven that the S protein of SARS-CoV-2 binds with 10−20-fold higher affinity to ACE2 than that of SARS-CoV (McKee et al. 2020). Thus, inhibition of ACE2 via a competing substance, seems to be a reasonable approach for preventing SARS-CoV-2 infections. Quercetin had potent inhibitory effects on ACE in vitro*,* and in vivo when tested in rat models (Actis-Goretta et al. 2006; Al Shukor et al. 2013; Guerrero et al. 2012; Nileeka Balasuriya and Vasantha Rupasinghe 2011). However, ACE inhibitors—which are standard therapeutics for the treatment of high blood pressure (Actis-Goretta et al. 2006), and may have a vital role in viral infections and pneumonia (Henry et al. 2018)—have not yet been shown to inhibit ACE2.

## Non-specific inhibition of SARS-CoV

### Flavones and flavonols

Baicalin, a flavone glycoside and major constituent of *Scutellaria baicalensis,* was found to have antiviral in vitro activity against SARS-CoV (12.5 to 25 µg/ml after 48 h; and 25 to 50 µg/ml after 72 h) when screened against 10 clinical isolates of SARS-CoV with foetal rhesus kidney-4 cells tested in a plaque reduction assay (Chen et al. 2004). For comparison, lopinavir—a protease inhibitor used against HIV infections, and which is currently in clinical trials for anti-SARS-CoV-2 infections—showed antiviral efficacy against SARS-CoV infected fetal rhesus kidney-4-cells at a concentration of 4 μg/mL, using the same assay (Chu et al. 2004). Baicalin is also a known inhibitor of HIV-1 reverse transcriptase (Li et al. 2000), and was shown to possess inhibitory potential against influenza in vitro and in vivo by acting as immune modulator promoting IFN-γ production in mice and human CD4 + and CD8 + T cells (Chu et al. 2015; Ding et al. 2014).

In general, some HIV protease inhibitors e.g., lopinavir, ritonavir or nelfinavir could interfere with SARS-CoV replication in-vitro, and had binding affinity to SARS-CoV 3CLpro (Zhang et al. 2004). Based on available evidence, it is controversial whether antiretroviral drugs can be suggested for the treatment of coronavirus diseases such as SARS-CoV-2. However, flavonoids which were found to have anti-HIV activity may be still worth investigating for their anti-SARS-CoV-2 potential. For example, baicalein, another major component from *Scutellaria baicalensis,* has been described for its anti-HIV properties such as its binding to HIV-1 integrase (Hu et al. 2010; Zhao et al. 2016), and thus might be proposed as interesting lead compound for SARS-CoV-2 drug discovery.

In addition to the inhibitory potency of kaempferol against SARS-CoV proteases (Table 2), the flavonol was able to block the 3a ion channel of SARS-CoV, a protein encoded by the open-reading-frame (ORF) 3a, which is involved in the virus release mechanism (Schwarz et al. 2011). Schwarz et al. demonstrated that glycosides of kaempferol are stronger inhibitors, highlighting the importance of sugar residues (Schwarz et al. 2014). The kaempferol glycoside juglanin having an arabinose residue, was shown to be the most prominent substrate (IC_50_ value = 2.3 µM) (Schwarz et al. 2014).

### Procyanidins

Procyanidins such as procyanidin A2, and procyanidin B1 isolated from Cinnamomi Cortex had inhibitory potential against wild-type SARS-CoV using a plaque reduction assay (IC_50_ = 29.9 and 41.3 µM, Table 2). However, it was reported that the interplay of all procyanidins in the extracts from Cinnamomi Cortex appeared to be crucial for causing strong activity against wild-type SARS-CoV (Zhuang et al. 2009). Plaque reduction assays do not indicate any specific inhibition mechanism of the compounds, but provide a fast and high throughput pre-screening method.

## Most promising flavonoids with anti-SARS-CoV-2 potential

Analyses of 17 studies based on flavonoids as anti-CoV agents revealed 47 compounds (Table 2) as possible agents against SARS-CoV-2. Among them, 16 compounds were investigated in at least two independent studies. 4 of these compounds, quercetin, herbacetin, isobavachalcone, and kaempferol, show activity against at least two viral targets. According to the high IC_50_ values of kaempferol (116.3 µM against SARS-CoV 3CLpro, and 206.6 µM against MERS-CoV PLpro), we do not consider this compound a promising candidate to treat SARS-CoV-2. Therefore, the flavonols, herbacetin and quercetin, along with isobavachalcone (Fig. 2) were identified to be the most attractive antiviral leads against SARS-CoV-2.—Due to their IC_50_ values and broad-spectrum activity against proteases of SARS- and MERS-CoV described in different studies (Table 2), as well as their availability from different plant sources. Given the similarity of the genomic sequences encoding the 3CLpro and PLpro catalytic sites of SARS-CoV-2, SARS-CoV, and MERS-CoV (Fig. 1), 3CLpro and PLpro inhibitors of SARS- and MERS-CoV are expected to be valuable leads for treating SARS-CoV-2 infections and COVID-19. For instance, remdesivir—one of the most important antiviral drugs which has been suggested for therapeutic use against SARS-CoV-2—showed broad-efficacy against both SARS- and MERS-CoV (Sheahan et al. 2017, 2020).

Based on the high CC_50_ values of the active flavonoids, such as that of herbacetin CC_50_ = 293.7 µM (Jeong et al. 2009) and quercetin CC_50_ = 385.5 µM (Chiow et al. 2016), they can be used in higher concentrations in therapeutic treatments without causing any substantial cytotoxic effects. Prenylated flavonoids like isobavachalcone, are reported be relatively non-toxic to non-cancer cells (Šmejkal 2014), and can be found in significant amounts in the plant families Moraceae, and Fabaceae (Kuete and Sandjo 2012).

As quercetin, herbacetin and isobavachalcone are readily available in high amounts from several plant sources, this might be also beneficial for future investigations on SARS-CoV-2, and the development of anti-CoV therapeutics.

Although the daily intake of plant food may appear to be a readily accessible source of active flavonoids, there is no scientific evidence that a high consumption of flavonoid-rich food and/or supplements, like that of quercetin, provides significant protection against viral diseases in humans e.g., against SARS-CoV-2. Due to low bioavailability, fast metabolism and elimination of flavonoids, the biological functions of these dietary polyphenols in-vivo are likely to be compromised. On the other hand, there are some external, non-host related, factors limiting the bioavailability of dietary flavonoids which include: environmental factors (i.e., storage, sun exposure), food processing factors (i.e., cooking), the interaction with other polyphenols present in plant-based food (i.e., antagonistic actions), the chemical structure (i.e., polymer or in glycosylated structure) and the concentration of the dietary compounds (D'Archivio et al. 2010).

Besides strong inhibitory effects of flavonols and chalcones on SARS- and MERS-CoV, these flavonoids also have some anti-inflammatory (Choy et al. 2019; Ur Rashid et al. 2019) and immune-modulating properties (Hosseinzade et al. 2019), which can be highly beneficial in the host immune response to viral infections. As an example, quercetin which is well-documented for its wide spectrum of antiviral functions (Zakaryan et al. 2017), had antiviral effects against rhinovirus (RV) infections in-vitro and in-vivo using RV-infected mice, by reducing the expression of pro-inflammatory cytokines and chemokines (Ganesan et al. 2012). Hence, due to different important functions they can be valuable for either preventing and treating viral infections, as well as for alleviating symptoms (e.g., fever, cough, etc.) that may occur during the course of viral infections like that of SARS-CoV-2.

In general, quercetin can be considered a highly potent candidate for treating SARS-CoV-2, as it has affinity to a variety of anti-CoV drug targets. The flavonol interfered with the viral replication of SARS-CoV by blocking the enzymatic activities of 3CLpro, PLpro (Table 2) and helicase (IC_50_ = 8.1 µM) (Lee et al. 2009), but also impaired SARS PLpro cleavage activity of ubiquitin and interferon-stimulated gene (ISG) 15 (IC_50_ = 20.7 and 34.4 µM, respectively) (Park et al. 2017), which can be important for both the viral replication and the host immune response to viruses (Cho et al. 2013). Moreover, quercetin interacted with SARS-CoV at host cell entry level by binding to the S glycoprotein (EC_50_ = 83.4 µM) that docks to the host receptor ACE2 (Zhou et al. 2020). Further, quercetin demonstrated proteolytic activity against MERS-CoV 3CLpro (IC_50_ = 34.8 µM, Table 2).

### Future perspective of flavonoids in therapeutic use

Although quercetin, herbacetin and isobavachalcone represent effective compounds against SARS and/or MERS-CoV in vitro, it does not directly indicate their in vivo efficiency or clinical use as anti-SARS-CoV-2 therapeutics. Further experimental in-vivo studies are suggested to evaluate their possible preclinical and clinical efficacy for the prevention of SARS-CoV-2 infections and the treatment of COVID-19.

For that purpose, there are a few issues that need to be addressed.

Since polyphenolic compounds are known for their aggregating tendency, doubts have been raised about the reliability of in vitro bioassay data of flavonoids, as false-positive results may occur through non-specific binding of phenolic compounds to proteins (Pohjala and Tammela 2012). The formation of aggregates can cause a non-specific inhibition by sequestering enzyme molecules, absorbing or adsorbing them within their structure and thus causing denaturation (Coan et al. 2009; Pohjala and Tammela 2012). Quercetin, which is one of the best studied flavonoid, showing virucidal activity against various enveloped viruses (e.g., influenza (Wu et al. 2015), parainfluenza type 3, herpes simplex (Chen et al. 2006)), was among the flavonoids that form large aggregates and thus may operate as a promiscuous inhibitor affecting various unrelated targets (Pohjala and Tammela 2012). However, it has been reported that the addition of Triton X-100, a solubilizing agent, to proteolytic assays, can help to reduce aggregate formation and complexation, such as that of flavonoids (Jo et al. 2020; Pohjala and Tammela 2012). Besides the limitations of the bioassays, there is another aspect—the in-vitro molecular assays and cell-based assays do not provide any information about the bioavailability of the compounds. However, this aspect is not within the scope of the review, but for developing flavonoids further, this aspect should be considered. Therefore, it is important to address the bioavailability of flavonoids as one of the critical limiting factors in their therapeutic use. Different strategies to enhance the stability, solubility and systemic distribution of flavonoids have been reported, which can be employed. These include nanotechnology, co-crystallization, absorption enhancers and structural transformations (e.g. prodrugs, glycosylation) (Ajazuddin and Saraf 2010; Zhao et al. 2019).

The use of the flavonoids quercetin, herbacetin, and isobavachalcone in adjuvant therapy with other proposed antiviral drugs may present another interesting approach to combat SARS-CoV-2 infections. Combination therapy often leads to better outcomes in antiviral treatments. For instance, it was found that synergistic effects between quercetin and the antiviral agent aciclovir resulted in enhanced antiviral activity against pseudorabies herpesvirus infection in-vitro (Ahmad et al. 2015). Similarly, α-glucoside inhibitors applied together with ribavirin, a standard antiviral drug, could improve antiviral efficacy against dengue infection in vitro and in vivo (Chang et al. 2011).

Furthermore, it should be also evaluated whether the pure flavonoid itself like quercetin, herbacetin, and isobavachalcone, or adminstered in combination with other flavonoids and/or natural compounds can achieve optimal health benefits.

The increasing development of drug-resistance in antiviral treatment might be another critical matter for the therapeutic use of flavonoids.

## Conclusions

**Fig. 3:**
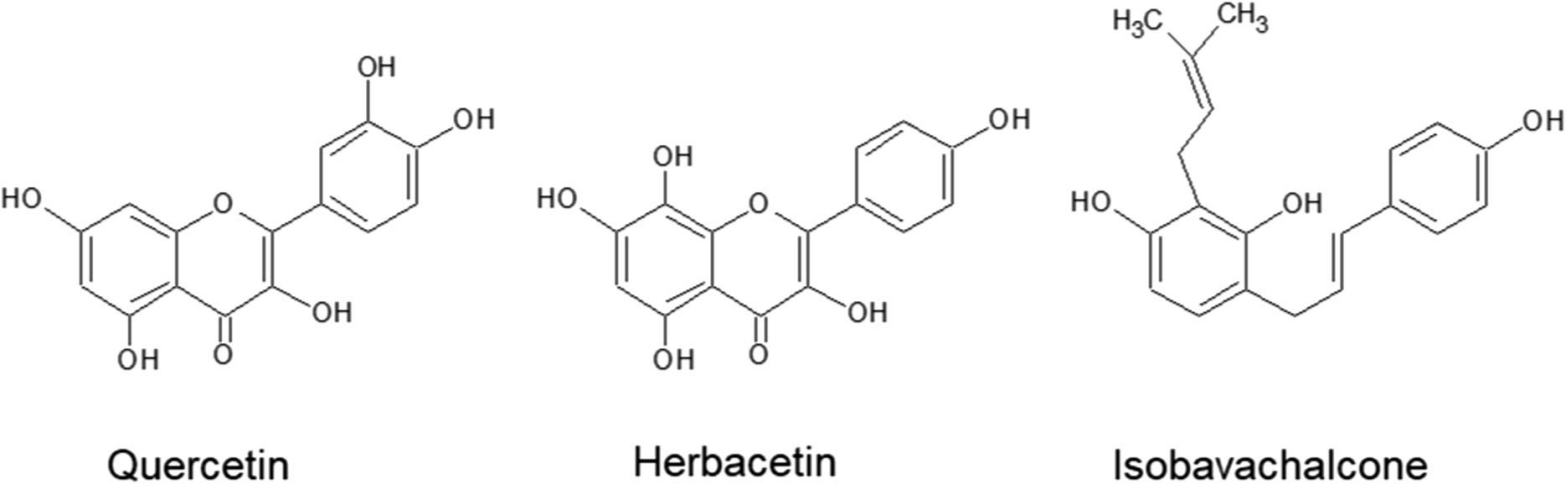
Chemical structures of the most promising flavonoids

Literature search in PubMed, has led to the identification of numerous flavonoids exerting antiviral in vitro activity against SARS and/or MERS coronavirus, primarily by inhibiting the enzymatic activity of 3CL and PL. Flavonols and chalcones were found to be the main groups of flavonoids containing the highest number of effective compounds against 3CL and PL proteases. In particular, herbacetin, quercetin and isobavachalcone (Fig. 3) were identified as promising antiviral leads against SARS- and MERS-CoV based on their broad-spectrum activity against the viral proteases 3CL and PL of both CoVs, the number of relevant literature data, and the availability of the compounds from different plant sources. Considering the fact that quercetin could interfere with SARS-CoV at different levels, such as at the viral entry and replication process, as well as with 3CL protease of MERS-CoV, we specifically propose this compound to be a highly valuable anti-SARS-CoV-2 candidate. Quercetin may prevent host cell entry of SARS-CoV through binding to the S protein and inhibiting ACE. Further, the flavonol can impair the viral replication of SARS- and MERS-CoV by blocking the enzymatic activites of 3CL and PL including SARS-helicase, and can inhibit the deubiquitination and deISGylation process of SARS PLpro, which might be also an issue in the host immune response. Other potent substrates able to interact with the enzymatic activity of SARS- and MERS-CoV proteases, were found to be prenylated flavonoids (e.g., isobavachalcone). However, despite some promising inhibitory activities of flavonoids against SARS- and MERS-CoV in vitro, none of these compounds have been tested in vivo using animal and/or human cell models. Therefore, more detailed investigations on pharmacological mechanisms, long-term toxicology, bioavailability, as well as some additional studies on possible herb-drug interactions are required. In fact, natural compounds like flavonoids, continue to be a wealthy source for the discovery of novel antiviral agents. It was reported that natural substrates from Traditional Chinese Medicine (TCM) seemed to have a positive impact on the recovery process of patients suffering from SARS-CoV diseases, by mitigating possible side effects of conventional therapeutics (Yang et al. 2020). Since flavonoids are well-documented for their broad spectrum of health-beneficial properties, especially anti-inflammatory and antioxidative effects, they could be of great value for strengthening the host immune response to viral diseases, and alleviating infection-related symptoms, such as down-regulating overwhelming inflammatory responses (e.g. cytokine production). For instance, besides the antiviral effects of quercetin, the flavonol was found to prevent tissue damage by scavenging free radicals, and could reduce the release of inflammatory cytokines such as interleukine IL-8 (Kinker 2014).

Due to high similarities of SARS-CoV, SARS-CoV-2, and MERS-CoV—sharing several homologous viral proteases, the proposed flavonols herbacetin and quercetin, as well as isobavachalcone may demonstrate attractive antiviral substrates against SARS-CoV-2 and/or other coronaviruses. However, further research and more detailed pharmacological investigations in-vivo, particularly on the bioavailability of these compounds, appear to be a promising approach for the discovery of novel herbal substrates used as adjunctive therapeutics in the treatment of coronavirus diseases, such as COVID-19. Furthermore, greater attention should be paid to combinatory effects of flavonoids, especially when used together with other standard antiviral drugs.
